# Spatial dynamics of TB within a highly urbanised Asian metropolis using point patterns

**DOI:** 10.1038/s41598-017-00081-3

**Published:** 2017-02-24

**Authors:** Sourav Das, Alex R. Cook, Win Wah, Khin Mar Kyi Win, Cynthia Bin Eng Chee, Yee Tang Wang, Li Yang Hsu

**Affiliations:** 1 School of Mathematics, University of Bristol, Bristol, UK; 2 Saw Swee Hock School of Public Health, National University of Singapore and National University Health System, Singapore, Singapore; 3 Tuberculosis Control Unit, Singapore TB Elimination Program, Ministry of Health, Singapore, Singapore

## Abstract

Singapore is a high-income country in a region with a high prevalence of tuberculosis. The Singapore Tuberculosis (TB) Elimination Program (STEP) was set up in 1997, and the better surveillance and clinical management practices initiated under STEP led to a decade-long decline in the incidence levels. However, incidence rates started to rise again since 2008. The reasons for this rise are unclear. This study involved a spatial analysis of the epidemiology of TB among Singapore residents. More than 30 000 cases reported during 1995–2011 and their residential addresses were analysed for spatial risk and spatial clustering, using spatial point pattern methodology. The principal factor responsible for the increasing resident TB incidence in Singapore is the changing age profile of the population. In particular the burgeoning population aged above 65 years accounts for the increase in reported cases. Singapore’s population has one of the world’s lowest fertility and mortality rates, and the elderly population is projected to grow substantially over the next few decades. Tuberculosis rates may therefore continue to rise even with static or improving case management and surveillance.

## Introduction

Tuberculosis (TB) has been a leading cause for death in human societies for decades. In 1993 the World Health Organization declared TB to be a global health emergency^1, 2^. Its reduction was later included as one of the challenges in the global Millennium developmental agenda^3, 4^ to be achieved by 2015^5^. In 1995 an annual surveillance program was launched under the auspices of the World Health Organization (WHO). During the past two decades there have been substantial accomplishments. In particular, the mortality rate has reduced by 47%^5^, and TB incidence is 18% lower than the levels of 2000. However, challenges remain. In its latest report^5^, the WHO notes that in 2014 an estimated 9.6 million fell ill with TB. Of these, 6 million cases were new patients. Together with HIV, TB continues to be a major cause of death (1.2 million reported deaths in 2014) globally.

Of the new cases, 58% are estimated to be in the South East Asia or Western Pacific regions. In this study we investigate the epidemiology of TB among the susceptible resident population of south-east Asia’s most economically advanced nation, Singapore, situated at the end of the Malay Peninsula. The city state’s location in one of the high TB burden zones of the world makes her vulnerable to tuberculosis. As the global urban population crossed 50% for the first time in 2014, largely driven by developing nations^6^, Singapore’s TB epidemiology may have important lessons for densely populated urban areas of high TB burden countries such as India (23% of global cases), China (10%) and Indonesia (10%).

Incidence rates among Singapore residents declined from 300 (per 100,000pa) in the early 1960s to 106 (per 100,000pa) in the mid-1980s^7–9^. In 1997, the Singapore Tuberculosis Elimination Programme (STEP)^7^ was launched to address a decade-long stagnation in TB rates at 50–55 (per 100,000pa) during 1987 to 1997. Thereafter, TB incidence rates among Singapore residents declined from 58 (per 100,000pa) in 1998 to a historic low of 35 (per 100,000pa) in 2006 and 2007. However, since 2008 the TB incidence has begun to rise to around 39 per 100,000 population per year. The reasons for this surge, which contrasts with global improvements, remain unclear since there has been no association study on the influence of the demographic characteristics on TB in Singapore.

In the present study, we analyse more than 30,000 cases reported between 1995 and 2011. The aim of this paper is twofold: first, to analyse spatial risk of TB prevalence in Singapore and investigate geographically structured factors among residents at planning area levels of the city state, developing spatial risk maps for public health interventions. Second, we assess demographic factors associated with the rise in reported TB incidence among Singapore residents. A detailed epidemiology of non-resident cases will be reported separately.

To this end, we apply statistical point pattern methodology, to the residential addresses of patients, to investigate the following. 1a. Does the spatial spread of TB indicate heterogeneity? 1b. Has the spatial trend changed significantly over time? If so which are the zones with higher prevalence? 2. What is the major demographic factor behind the increase of TB among resident Singaporeans? 3. Are there indications of transmission between residents and non-residents? Non-resident cases (and their residential addresses used here) exclude individuals who have lived in Singapore for less than three months. This censors short term visitors and medical tourists.

## Results

**Figure 1:**
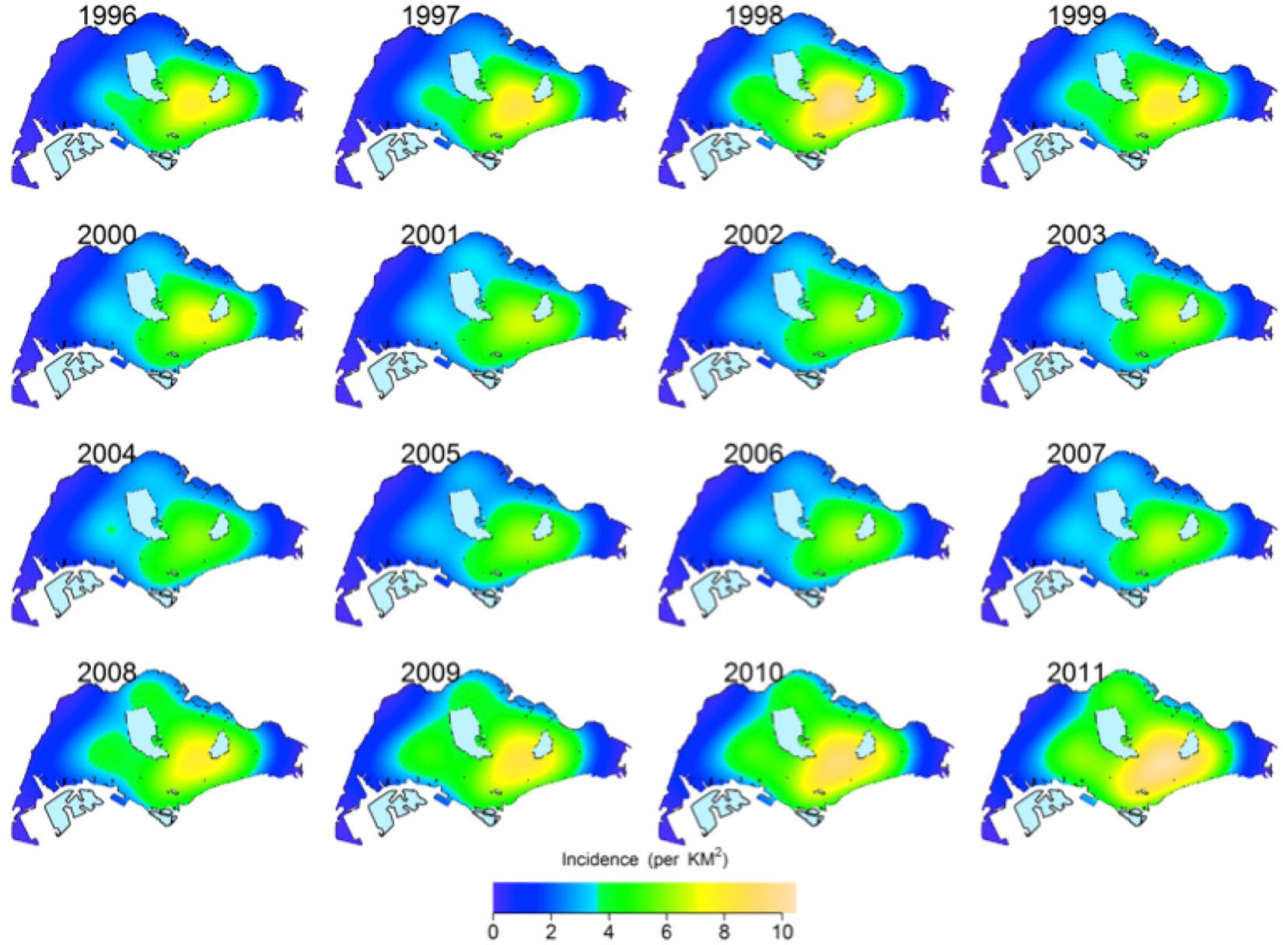
Interpolated annual spatial intensity maps of TB risk in Singapore based on all reported cases. Purple implies lowest intensity and dark yellow signify high intensity areas. Plots were created in statistical program R^22^ using the package *spatstat* ^19^. Administrative and national political coordinates of Singapore were provided, for academic research and publication, by the Singapore Land Authority (SLA)^24^.

The estimated spatial intensity of TB cases across Singapore is presented in Fig. 1, indicating substantial spatial heterogeneity across the island and the evolution of spatial incidence with time. The intensity of TB cases increased in the east and southern parts of the city, between 1995 and 1999, followed by a period of decline during 2000–2007 and showed a resurgence between 2008 and 2011.

**Figure 2:**
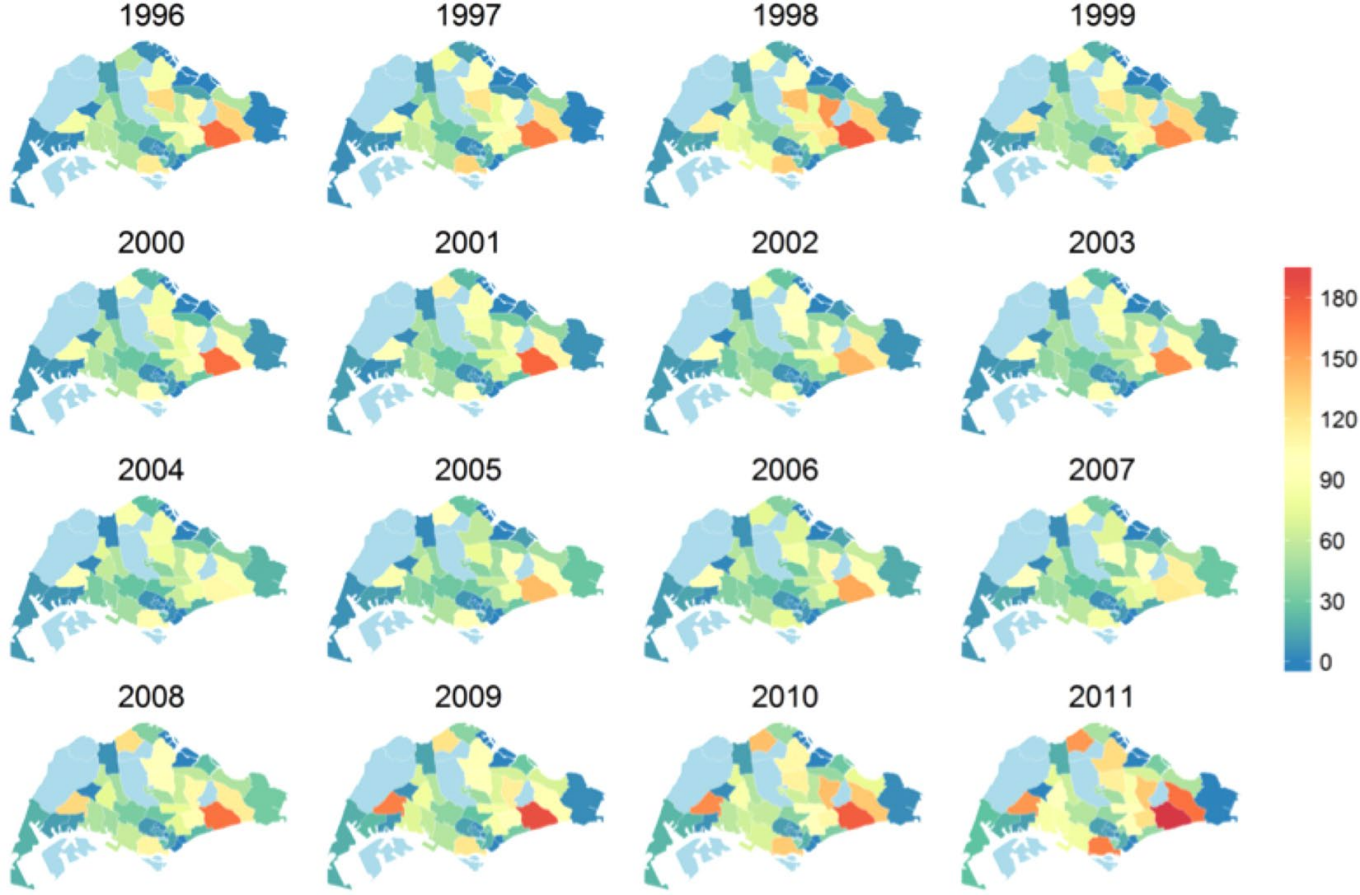
Estimated case count of TB cases in 44 planning areas of Singapore. Counts were computed by integrating the estimated spatial intensity over the planning area polygons. The plots capture higher risk of cases in Bedok and the surge of cases in Jurong West. Plots were created in statistical program R^22^ using the package *spatstat* ^19^. Administrative and national political coordinates of Singapore were provided, for academic research and publication, by the Singapore Land Authority (SLA)^24^.

Figure 2 shows the spatial intensity map of TB at the planning area level. A few specific planning areas have noteworthy patterns: Bedok in the south east has consistently featured as the area with highest TB risk, followed by Tampines and Hougang, also in the east. Woodlands in the north and Bukit Merah in the south also have relatively high disease intensity, while Jurong West has seen a sharp increase in TB incidence.

**Figure 3:**
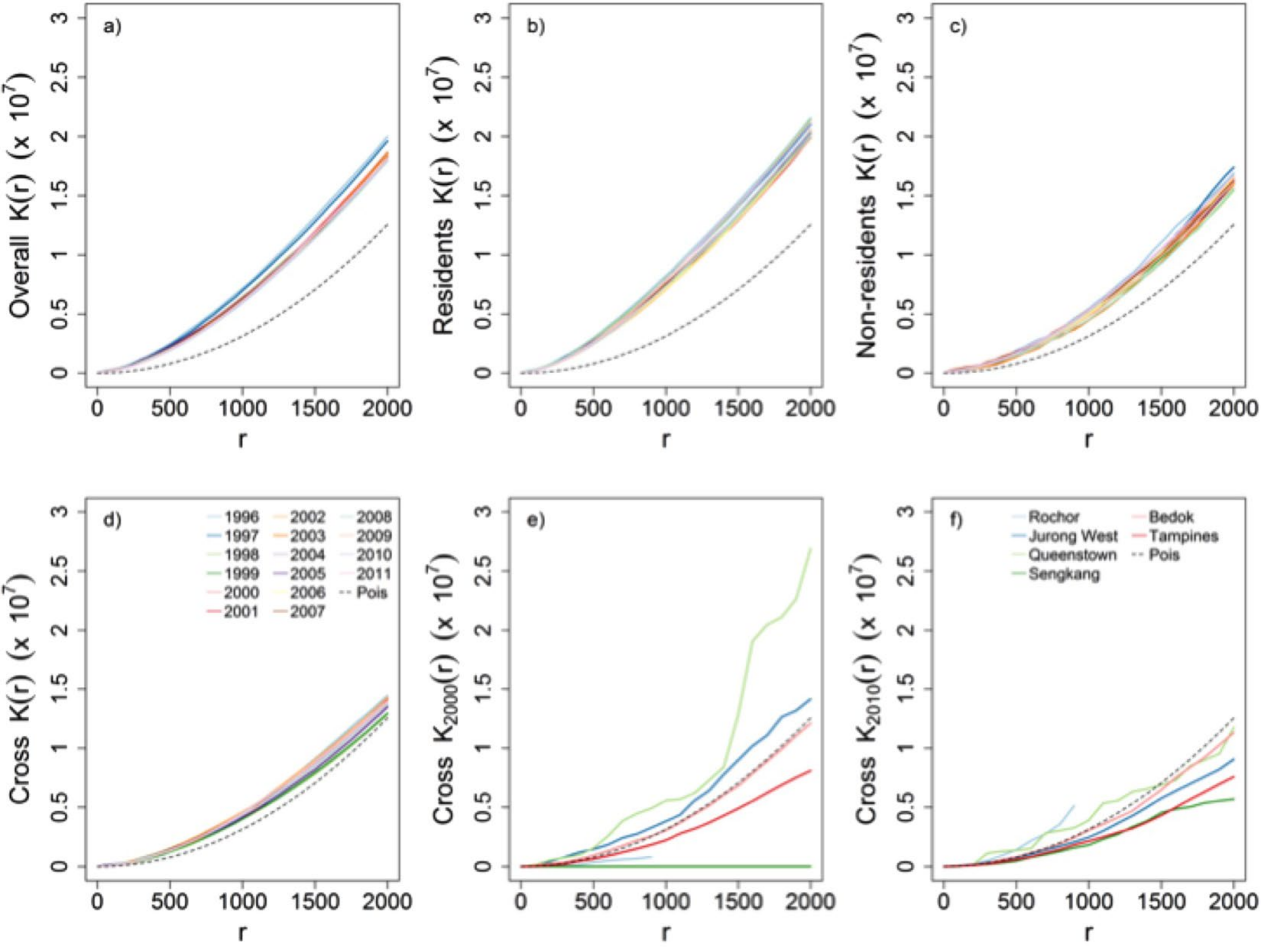
*K-function* plots studying clustering phenomena among various categories of reported TB cases compared with completely random spatial pattern (dashed line). (**a**) Annual spatial clustering curves combining all TB cases over seventeen years from 1996 to 2011, (**b**) Annual spatial clustering patterns of resident TB cases observed over the same time period, (**c**) Annual spatial clustering patterns among non-resident reported TB cases during 1996–2011, (**d**) Annual spatial clustering curves studying clustering of non-resident TB cases around resident TB cases reported during 1996–2011, (**e**) Spatial clustering curves studying clustering of resident and non-resident reported TB cases in three *high* TB (Jurong West, Bedok and Tampines) zones and *low* TB zones in 2000, (**f**) Spatial clustering curves showing clustering of resident and non-resident reported TB cases in three *high* TB (Jurong West, Bedok and Tampines) zones and *low* TB zones in 2010.

The spatial clustering of TB, as measured via the inhomogeneous *K*-function, is presented in Fig. 3, stratified by year. The *K* function was separately plotted for *all* cases as well as for two demographic constituents, residents and non-residents. Figure 3 also presents inter-group (resident cases in the neighbourhood of non-resident cases) clustering using the cross-*K*_*c*_(.) function.

Within each demographic grouping it can be seen that TB case residences are more clustered than they would be due to chance alone, assuming a heterogeneous intensity function, *λ*(*s*), for all distances considered up to 2 km. This may reflect heterogeneity in underlying risk due to differences in age structure or the environment, for example, or be due to stochastic clustering; as noted by Bartlett^10^ these cannot be distinguished using a single point pattern.

When all cases are considered together, in the top left panel, we note that the extent of clustering decreased from the 1990s to the later 2000s, indicating that while the incidence was generally higher in 1998 than in 2009 (Fig. 1), cases became less clustered over time. The cross-*K*_*c*_(.) function plot does not indicate significant residential clustering of resident cases around non-resident cases or vice-versa, suggesting that disease among the two groups is close to being independent. Figure 3 also presents the cross K function between residents and non-residents in 2000 and 2010 for three zones with high (Bedok, Jurong West, Tampines) and low (Queenstown, Rochor, Sengkang) TB incidence. There is no evidence of clustering between the two demographic groups, with the cross K function indicating that cluster levels are close to what would be expected by two processes that are either independent or have a slight negative (i.e. repulsive) relationship.

**Figure 4:**
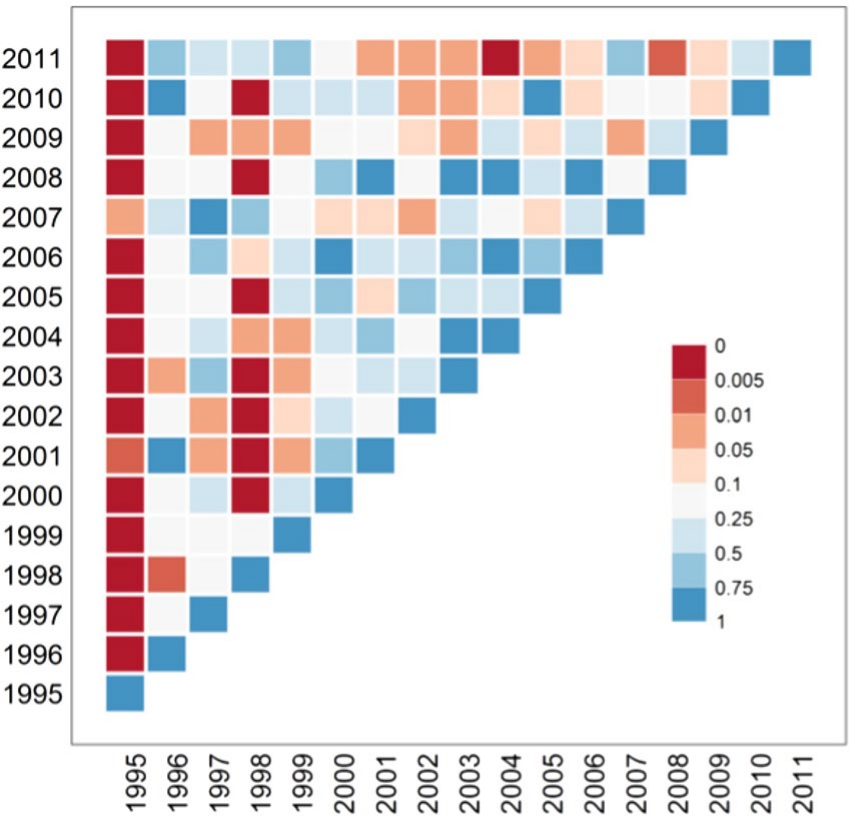
Matrix of *p* values from Hotelling’s T^2^ tests. Under H_0_ the T^2^ statistic follows a central F distribution. The test is described in the Methods section. We compare the incidence rates of TB among resident Singaporeans, for planning areas of Singapore for a pairs of years. The colour *red* implies significant statistical difference in overall spatial risk between the two years while *blue* implies no difference.

Figure 4 shows the *p*-values for all distinct pairs of years using the spatial Hotelling’s *T* ^2^ test, to compare resident TB incidence rates (per 10,000) at planning area levels. From 1996 to 2011 the *p*-value matrix displays a roughly uniform distribution, with only a slight skew towards significant differences: 15% of *p*-values are significant at the 5% and 5% at the 1% level, implying there is little evidence of a change in the population adjusted annual TB incidence among residents. Note that in 1995 – the first year of residential address based notification – many cases remained unreported, and this year has most of the significant differences present in this analysis. This has led to statistically significant difference of annual incidence with all other years, across planning areas. This is shown in Supplementary Figure 1 shows this.

**Figure 5:**
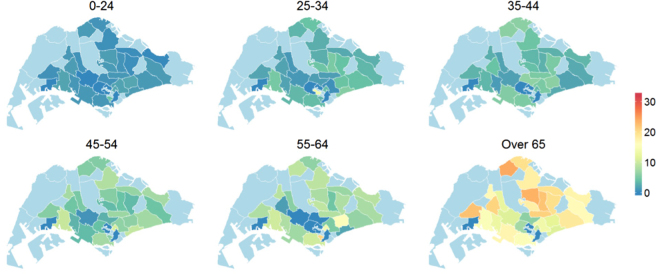
Age and zonal distribution of TB among resident Singaporeans in the year 2000. The values are adjusted for the population size and expressed per 10,000 of the susceptible population. Plots were created in statistical program R^22^ using the package *spatstat* ^19^. Administrative and national political coordinates of Singapore were provided, for academic research and publication, by the Singapore Land Authority (SLA)^24^.

**Figure 6:**
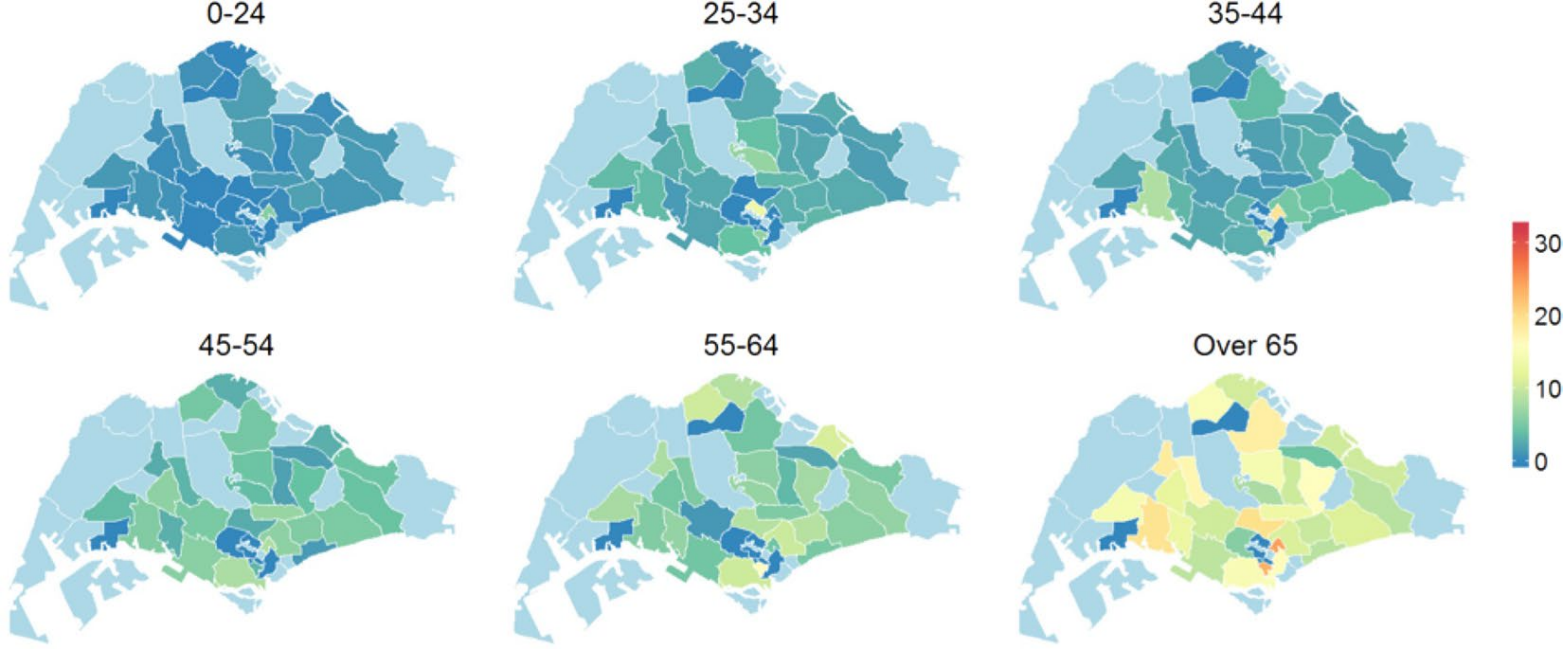
Age and zonal distribution of TB among resident Singaporeans in the year 2010. The values are adjusted for the population size and expressed per 10,000 of the susceptible population. Plots were created in statistical program R^22^ using the package *spatstat* ^19^. Administrative and national political coordinates of Singapore were provided, for academic research and publication, by the Singapore Land Authority (SLA)^24^.

Figures 5 and 6 show the reported incidence rates of TB among residents, in Singapore, over six different age groups in 2000 and in 2010, respectively. The figures indicate that, for Singapore residents, incidence is highest in the age group of over 65 year olds, and that incidence within this age group fell from 20–30 reported TB cases (per 10,000 population) in 2000 to below 15 cases (per 10,000) in most planning areas, including all major planning areas such as Bedok, Bukit Merah and Jurong West.

**Figure 7:**
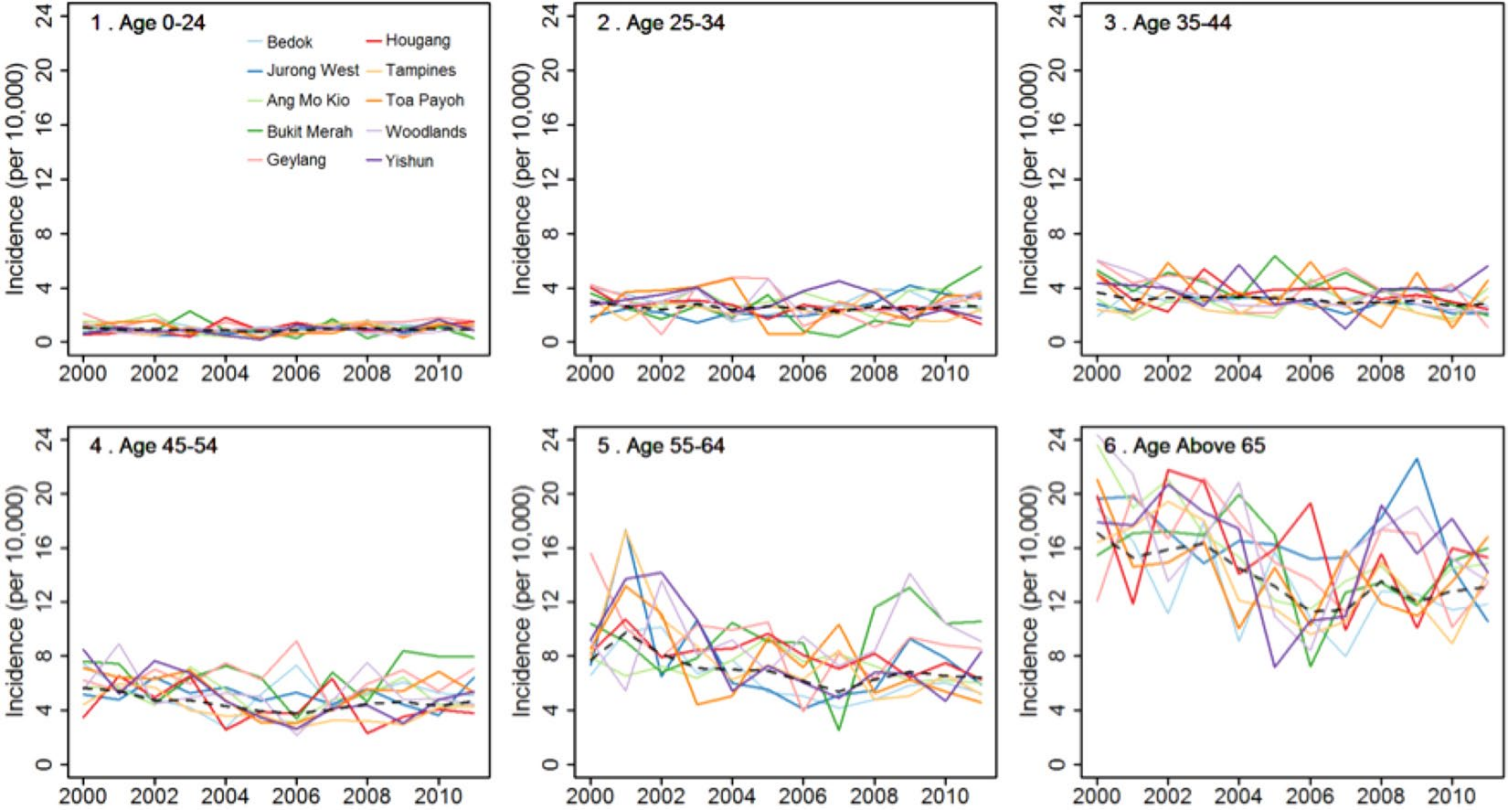
Resident annual time series incidence for ten leading planning areas of Singapore across six different age groups from 2000 to 2010. The incidence is adjusted for underlying susceptible population.

In Fig. 7 we show the annual time series of resident TB cases from 2000 to 2010 for the six age groups, adjusted for population, across ten major planning areas. Together with Figs 5 and 6 the panels of Fig. 7 corroborate two principal factors: firstly there is a clear gradient of TB incidence from lowest levels (close to 0 per 10,000) among the youngest age group 0–24 to highest levels (of above 16 cases per 10,000) the senior age group of above 65. Secondly, on adjusting for susceptible population the annual trends of TB incidence for almost all planning areas remain stable across all age groups.

## Discussion

TB incidence in Singapore is lower than in neighbouring countries, but higher than other countries with similar levels of economic development, and incidence rates have been rising despite earlier successes^9^. In an earlier paper, we had investigated the temporal evolution of TB incidence in Singapore^11^. In this study, using more than 22,000 cases reported during 1995–2011, we have analysed the spatial variation of TB incidence across the city state, among residents. The period 1995–2002 saw cases concentrated in the south eastern planning areas of Singapore. Bedok consistently had the highest concentration of around 10 cases per square km with high to medium case density (between 4–8 cases per square km) in the surrounding areas of Tampines, Hougang and Ang Mo Kio. The western planning areas had low spatial risk, reflecting the relatively lower residential density in these parts during that time. Count of reported cases and the spatial extent of distribution of cases had peaked during 1998. Since 1999 the case count and spatial range of cases had started declining, simultaneously. These coincided with the implementation of new policies on clinical and epidemiological surveillance under the Singapore TB elimination program^9^. During 2003–2007 the annual case count continued to remain low manifesting similar patterns of spatial variation, each year. South eastern planning areas had spatial risk of 4–6 cases per square km while rest of the city state had a low spatial intensity of 0–2 cases per km square. The period of 2008–2011 is marked by increasing annual case count together with rising spatial intensity in all administrative zones. The annual case count had started rising from its lowest levels of 1989 cases during 2002 to the highest level of 3240 in 2011. Residential development in the western planning areas also led to the unprecedented spatial risk of cases in the western planning areas.

The spatial intensity maps show that the prevalence of TB is clearly heterogeneous, with zones in the south and south east having a higher intensity of cases than most of the west of the city state. These zones are also the more established residential areas. However, the fast changing urban landscape, with increasing development of the west to accommodate a population that has increased from 3 to 5 million from 1990 to 2010, has been associated with more TB cases reported from the western planning areas. Second order spatial point pattern analyses showed strong clustering of residential address among both resident and non-resident communities. But we did not find evidence of significant residential clustering of non-resident cases in neighbourhoods with high resident incidents, indicating low probability of inter-group transmission in residential neighbourhoods. This however does not discount other forms of prospective transmission at places of occupation, business and transportation networks, an area that should be investigated in future studies.

The lack of evidence of clustering among local resident cases around foreign cases suggests that other factors are responsible for the increasing incidence of TB among residents. We found that the spatial structure of planning area level susceptible population was the primary explanatory factor underpinning spatial variation of case incidence during the time horizon of the study (1995 to 2011). It was observed that the population annual incidence rates did not show statistically significant temporal variation.

We also discovered that after stratifying by age group, at both the national and regional level, age-specific incidence among residents actually fell (in contrast to the non-age stratified incidence, which rose) over the time course of the study. One cause of the increase at the national level can therefore be attributed to the aging of the population—which, due to the low fertility and mortality rates of Singapore residents is expected to accelerate to 2050^12^—which has led to more individuals being of an age in which reactivation of latent TB (acquired perhaps during the 1930s to 1960s, a time when Singapore was yet to undergo its transformation from low to high income), may be more likely^13^.

Singapore’s resident TB epidemiology is particularly relevant for other Asian countries that are developing and aging rapidly, such as China. Much like the city state, China has undergone rapid and extensive urbanization in the past three decades. China also features prominently in global terms for both TB (10% of the global cases) and aging (China being home to 23% of the global population of above 60 year olds and above^14^), and the growth of the latter may spur that of the former, as in Singapore.

The primary limitation of the study is that the denominator for the non-resident population is not known for Singapore, precluding incidence rates for this segment of the overall population. It is known that the size of the non-resident population has risen over the time period being studied, and now stands at 29% of the total population^15^, but the breakdown by age, or nationality, is not available. As a result, a full analysis of the spatial incidence rates for non-residents, matching what we did for residents, was not possible. However, the analysis of the spatial correlations between resident and non-resident cases indicated near-independence of the two groups, so the scant information on the non-resident population does not prevent a thorough assessment of the resident population.

## Conclusion

In this study, spanning 17 years of reported TB cases, we found that the major risk factor contributing to the recent rise in TB cases among Singapore residents is the burgeoning elderly population. Regions in the south and east of the city have relatively higher spatial risk due to the age distribution of susceptible population. TB incidence rates have declined among all age groups of the resident population in all areas of the city state. Reported cases display statistically significant spatial clustering within residents and non-residents, but there was no evidence of clustering between these two groups.

## Methodology

### Data

Under the Infectious Disease Act in Singapore, all suspected and confirmed TB cases are notified to the STEP registry of the TB Control Unit (TBCU) within 72 hours of starting TB treatment and/or laboratory confirmed results. The TBCU is the national unit for treatment of TB patients, contact investigation, preventive therapy, and educational training of health care workers in Singapore. We extracted data from the TBCU on all TB cases reported to the Singapore TB Elimination Program between 1995 and 2011.

Population data were obtained from Singapore Department of Statistics^15^ and Ministry of Health^8^. In particular we used planning area level population count categorized into five year age groups.

To map the spatial risk of epidemic we use spatial point process methodology. Heuristically, *events* occurring in a bounded spatial zone form a realisation of a point process if the number of location of these events, in this context, TB cases within the spatial extent of Singapore, are random processes. Realisations of a point process are called point patterns.

We define location of the *i*th TB case by (*x*_*i*_, *y*_*i*_) in the geographical window that spans the main island of Singapore, Pulau Ujong. The point pattern, *M* = {(*x*_1_, *y*_1_), (*x*_2_, *y*_2_) …, (*x*_*N*_, *y*_*N*_)}, is the set of all TB cases observed during a time period *T*.

We estimate first and second order moments (analogous to the mean and variance) of this point process, describing the spatial variation in risk and clustering of TB.

### Intensity function *λ*(.)

The intensity function, *λ*, at location (*x*, *y*) give the probability of observing a case in an infinitesimally small neighbourhood of (*x*, *y*), and the map of *λ*(.) over the entire spatial window represents the *spatial risk* of the event.

Mathematically *λ*(.) is defined as

1 $$\lambda (x,y)=\mathop{lim}\limits_{\{| dx| | dy| \to 0\}}(\frac{E[M(dx\,dy)]}{| dx| | dy| })$$

where *E*(.) denotes the expectation. For a completely random spatial point pattern (CSR), *λ*(.) measures the number of events per unit area. Consequentially, ${\int }_{A}\lambda (x,y)dx\,dy$ gives the expected number of events in an area *A*. An estimate of *λ*(*x*, *y*) is given by

2 $$\hat{\lambda }(x,y)=e(x,y)\sum _{i=1}^{n}\kappa ({x}_{i}-x,{y}_{i}-y)$$

where *κ*(·) is a bivariate Gaussian kernel with bandwidth selected by Diggle’s cross-validation^16^, and *e*(·) is an edge correction, specifically the translation correction^17^ to account for the reduced effective neighbourhood size near the edges of the chosen spatial domain.

Intensity maps were created by dividing the island into ~16 000 square cells of approximately 300 m in length, and evaluating the estimated intensity function thereon.

### *K*(.) function

The *K*(.) function, which measures interdependence of events and acts as a surrogate for transmission of disease and/or the influence of environmental factors, is defined to be

3 $$K(r)=\frac{E(r)}{\lambda }$$

where *E*(*r*) is the mean number of events within *r* spatial units of another event.

Thus *K*(*r*) is a scaled measure of the additional number of events in a neighbourhood of radius *r* of a case, given the spatial risk. For a completely spatially random process (CSR)—with a constant intensity function—the *K*(.) function at a distance *r* is *πr* ^2^ ^16, 18, 19^. The theoretical *K*(.) function for an inhomogeneous spatial Poisson process with intensity function *λ*(*s*) is also *πr* ^2^.

The modified cross-*K*(.) function^19, 20^ extends the *K*(.) function to *two* spatial point processes observed in the same window. That is for two different point processes *M*_1_ and *M*_2_, the cross-${K}_{c{M}_{1}{M}_{2}}(.)$ is a scaled average of additional number of events of *M*_2_ around an arbitrary event of *M*_1_. In particular, we calculate the cross K function to quantify the spatial interaction between cases of TB in the resident (*M*_1_) and non-resident (*M*_2_) populations.

The non-parametric unbiased estimate of *K*(.) function is given by^18, 19^

4 $$\hat{K}(r)=\frac{| A| }{{n}^{2}}\sum _{i=1}^{n}\sum _{j\ne i}{w}_{ij}I({r}_{ij}\le r)$$

where *r*_*ij*_ is the distance between events *i* and *j* and *w*_*ij*_ is the edge correction^21^. We compute the *K*(.) function for the overall TB population and separately for resident and non-resident population. If the estimated *K*-function $\hat{K}(r)$ is significantly higher than *πr* ^2^ at distance *r* then the data demonstrates aggregation or clustering indicative of transmission among infected individuals or of shared environmental factors. On the other hand, a value of $\hat{K}(r)$ significantly lower than *πr* ^2^ indicates ‘repulsion’ between the two sets. All *K*(.) curves were estimated under the assumption of inhomogeneous intensity function and compared against an inhomogeneous Poisson process. We used the function, Kinhom(.), of the spatstat package^19^ in software R^22^.

### Pairwise comparison of annual spatial incidence rates

We adapted Hotelling’s two sample T^2^ test^22^ to assess the hypothesis whether there were significant temporal variation in the annual spatial incidence of TB, among residents in Singapore. To compare we adjusted the case count at planning area levels to per 10,000 of the susceptible population. Let us define *K* to be the number of planning areas, *T*_1_ and *T*_2_ to be the two years to compare, and ${Y}_{k}^{T}$ to be the *adjusted resident case count* (where the adjustment is obtained by scaling the annual incidence per 10,000 of the planning area population) in the *k* ^*th*^ planning area in year *T*. The total adjusted resident case count in year *T* we denote ${N}_{T}={\sum }_{k=1}^{K}{Y}_{k}^{T}$.

Then, assuming that individual cases arrive randomly in time (for each year) and independently in space (among any of the *K* planning areas), we have,

5 $${{\boldsymbol{Y}}}^{T}={({Y}_{1}^{T},{Y}_{2}^{T},\ldots ,{Y}_{K}^{T})}^{^{\prime} } \sim Multinomial[{N}_{T},{{\boldsymbol{p}}}^{T}={({p}_{1}^{T},{p}_{2}^{T},\ldots ,{p}_{K}^{T})}^{^{\prime} }]$$

where ${p}_{k}^{T},k=1,2,\ldots ,K,$ are the population distribution of resident cases of the *k* ^*th*^ planning area in *T* ^*th*^ year.

***Y*** ^*T*^ can alternatively be expressed as the sum of *N*_*T*_ independent and identically distributed incidence vectors^23^ of order *K*, ${{\boldsymbol{X}}}_{i}^{T}=({X}_{i1}^{T},{X}_{i2}^{T},\ldots ,{X}_{iK}^{T})^{\prime}$, where ${X}_{ik}^{T}=1$ and ${X}_{i(k\ne l)}^{T}=0,$ for *i* = 1, 2, …, *N*_*T*_. This formally implies that the *i* ^*th*^ case was reported at the *k* ^*th*^ planning area and that an individual case can’t be present in two planning areas (or the boundary) at the same time. Then

${{\boldsymbol{X}}}_{i}^{T} \sim Multinomial[1,{{\boldsymbol{p}}}^{T}=({p}_{1}^{T},{p}_{2}^{T},\ldots ,{p}_{K}^{T})^{\prime} ],i=1,2,\ldots ,{N}_{T}$. Also, ${\boldsymbol{\Sigma }}={\rm{Var}}({{\boldsymbol{X}}}_{i}^{T}),$ is defined by

$$Cov({X}_{ik}^{T},{X}_{il}^{T})=(\begin{matrix}{p}_{k}^{T}(1-{p}_{k}^{T}),k=l\\ -{p}_{k}^{T}{p}_{l}^{T},k\ne l\end{matrix}).$$

Thus, ${{\boldsymbol{Y}}}^{T}={\sum }_{i=1}^{{N}_{T}}{{\boldsymbol{X}}}_{i}^{T}$. Since ${{\boldsymbol{X}}}_{i}^{T}$ are independently and identically distributed, for sufficiently large *N*_*T*_ using the central limit theorem, for a sample of random vectors, we have

6 $${\bar{{\boldsymbol{X}}}}^{T}\mathop{\to }\limits^{D}{N}_{k}({{\boldsymbol{p}}}^{T},{\boldsymbol{\Sigma }}/{N}_{T}),as\,{N}_{T}\to \infty .$$

### Test Statistic

Under the above framework if ${H}_{0}:{{\boldsymbol{p}}}^{{T}_{1}}={{\boldsymbol{p}}}^{{T}_{2}}$ and we define

7 $${t}^{2}=\frac{{N}_{{T}_{1}}{N}_{{T}_{2}}}{{N}_{{T}_{1}}+{N}_{{T}_{2}}}({\bar{{\boldsymbol{X}}}}^{{T}_{1}}-{\bar{{\boldsymbol{X}}}}^{{T}_{2}})^{\prime} {\hat{{\rm{\Sigma }}}}^{-1}({\bar{{\boldsymbol{X}}}}^{{T}_{1}}-{\bar{{\boldsymbol{X}}}}^{{T}_{2}}),$$

then

8 $${T}^{2}=\frac{{N}_{{T}_{1}}+{N}_{{T}_{2}}-K-1}{({N}_{{T}_{1}}+{N}_{{T}_{2}}-2)K}{t}^{2} \sim F(K,{N}_{{T}_{1}}+{N}_{{T}_{2}}-K-1),{\rm{under}}\,{H}_{0}.$$

Here, ${\bar{{\boldsymbol{X}}}}^{{T}_{j}}=\frac{1}{{N}_{{T}_{j}}}{\sum }_{i=1}^{{N}_{{T}_{j}}}{{\boldsymbol{X}}}_{i}^{{T}_{j}}$ and ${\widehat{\Sigma }}_{{T}_{j}}=\frac{1}{{N}_{{T}_{j}}-1}{\sum }_{i=1}^{{N}_{{T}_{j}}}({{\boldsymbol{X}}}_{i}^{{T}_{j}}-{\bar{{\boldsymbol{X}}}}^{{T}_{j}})({{\boldsymbol{X}}}_{i}^{{T}_{j}}-{\bar{{\boldsymbol{X}}}}^{{T}_{j}})^{\prime}$ where *j* = 1, 2. Also, $\hat{{\rm{\Sigma }}}=\frac{{N}_{{T}_{1}}{\hat{{\rm{\Sigma }}}}_{{T}_{1}}+{N}_{{T}_{2}}{\hat{{\rm{\Sigma }}}}_{{T}_{2}}}{({N}_{{T}_{1}}+{N}_{{T}_{2}}-2)}$, the pooled variance.

For the given samples we reject *H*_0_ at level of significance *α* if

9 $${T}^{2} > F(\alpha ;K,{N}_{{T}_{1}}+{N}_{{T}_{2}}-K-1).$$

Alternatively we reject *H*_0_ if the p-value,

10 $$p={P}_{{H}_{0}}[{T}^{2} > {T}_{Observed}^{2}] < \alpha .$$

We computed p-values corresponding to Hotelling’s *T* ^2^ for all $$(\begin{matrix}17\\ 2\end{matrix})$$ distinct pairs (1995 to 2011) of annual TB incidence rates, among Singapore’s residents. The results are shown in Fig. 3.

## Electronic supplementary material

Supplementary Data
