# Supplementary material for: Spatial dynamics of TB within a highly urbanised Asian metropolis using point patterns
Source: Sci Rep. 2017 Feb 24;7:36. doi: 10.1038/s41598-017-00081-3 (PMC5428374; doi:10.1038/s41598-017-00081-3)
Supplement: Supplementary file 1 — Supplementary Data [file 41598_2017_81_MOESM1_ESM.pdf]

Spatial dynamics of TB within a highly urbanised Asian metropolis using point patterns

Sourav Das*, Alex R. Cook†, Win Wah†, Khin Mar Kyi Win**, Cynthia Bin Eng Chee**, Yee Tang Wang**, Hsu Li Yang†**‡

*School of Mathematics, University of Bristol.

† Saw Swee Hock School of Public Health, National University of Singapore and National University Health System.

** Tuberculosis Control Unit, Singapore TB Elimination Program, Ministry of Health, Singapore.

‡corresponding author: hsuliyang@gmail.com

Supplementary Information

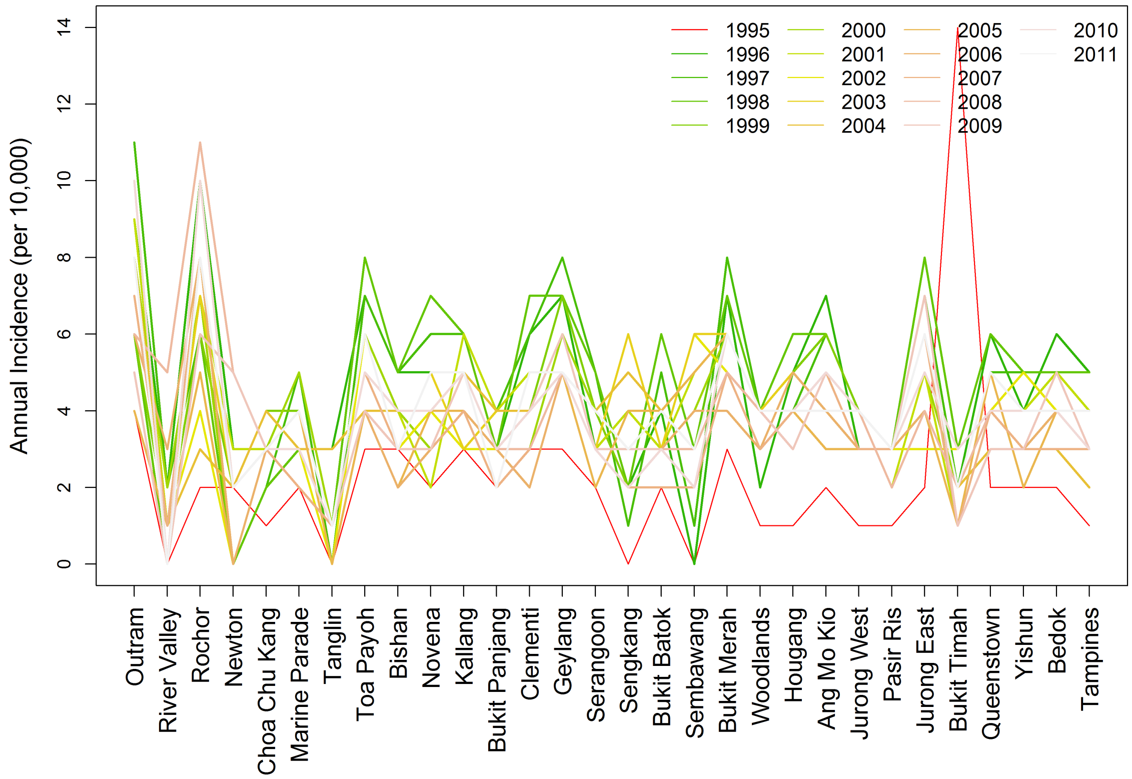

Figure S.1: Figure shows Annual incidence (per 10,000) of resident TB across thirty planning areas of Singapore between 1995 and 2011. Planning areas (x-axis) are presented in increasing order of land area.
